# Genetic characteristics of Korean Jeju Black cattle with high density single nucleotide polymorphisms

**DOI:** 10.5713/ajas.19.0888

**Published:** 2020-08-21

**Authors:** M. Zahangir Alam, Yun-Mi Lee, Hyo-Jung Son, Lauren H. Hanna, David G. Riley, Hideyuki Mannen, Shinji Sasazaki, Se Pill Park, Jong-Joo Kim

**Affiliations:** 1 Department of Biotechnology, Yeungnam University, Gyeongsan 38541, Korea; 2 Department of Genetic Engineering and Biotechnology, Shahjalal University of Science and Technology, Sylhet 3114, Bangladesh; 3 Department of Animal Sciences, North Dakota State University, Fargo, ND 58105, USA; 4 Department of Animal Sciences, Texas A&M University, College Station, TX 77843, USA; 5 Graduate School of Agricultural Science, Kobe University, Kobe 657-8501, Japan; 6 Faculty of Biotechnology, Jeju National University, Jeju 13557, Korea

**Keywords:** Jeju Black Cattle, Hanwoo, Genetic Diversity, Population Structure, Single Nucleotide Polymorphism (SNP) Chip

## Abstract

**Objective:**

Conservation and genetic improvement of cattle breeds require information about genetic diversity and population structure of the cattle. In this study, we investigated the genetic diversity and population structure of the three cattle breeds in the Korean peninsula.

**Methods:**

Jeju Black, Hanwoo, Holstein cattle in Korea, together with six foreign breeds were examined. Genetic diversity within the cattle breeds was analyzed with minor allele frequency (MAF), observed and expected heterozygosity (H_O_ and H_E_), inbreeding coefficient (F_IS_) and past effective population size. Molecular variance and population structure between the nine breeds were analyzed using a model-based clustering method. Genetic distances between breeds were evaluated with Nei’s genetic distance and Weir and Cockerham’s F_ST_.

**Results:**

Our results revealed that Jeju Black cattle had lowest level of heterozygosity (H_E_ = 0.21) among the studied taurine breeds, and an average MAF of 0.16. The level of inbreeding was −0.076 for Jeju Black, while −0.018 to −0.118 for the other breeds. Principle component analysis and neighbor-joining tree showed a clear separation of Jeju Black cattle from other local (Hanwoo and Japanese cattle) and taurine/indicine cattle breeds in evolutionary process, and a distinct pattern of admixture of Jeju Black cattle having no clustering with other studied populations. The F_ST_ value between Jeju Black cattle and Hanwoo was 0.106, which was lowest across the pair of breeds ranging from 0.161 to 0.274, indicating some degree of genetic closeness of Jeju Black cattle with Hanwoo. The past effective population size of Jeju Black cattle was very small, i.e. 38 in 13 generation ago, whereas 209 for Hanwoo.

**Conclusion:**

This study indicates genetic uniqueness of Jeju Black cattle. However, a small effective population size of Jeju Black cattle indicates the requirement for an implementation of a sustainable breeding policy to increase the population for genetic improvement and future conservation.

## INTRODUCTION

Cattle are an integral part of animal agriculture since 8000 BC, when it is thought that they become domesticated in different parts of the world such as India, Middle East and North Africa [1]. Different cattle breeds have been domesticated and adapted throughout the world due to variable geographical and climatic conditions. Jeju Black cattle (JJBC; Jeju Heugu) is one of the indigenous cattle breeds in the Jeju Island, south of Korean peninsula. The JJBC are thought to be originated from the native cattle in Korea main land according to island model of speciation [2]. The evidence of ancient cattle bones from the archaeological sites in Jeju Island suggests the existence of the breed approximately 1,100 to 2,000 years ago. DNA analysis of bones recovered from Gonaeri and Gwakji-ri in Aewaleup, Jeju city, indicates that ancestors of the present JJBC had been raised by humans since prehistoric times [3]. Also, the historical documents (Annals of the Choseon Dynasty) and the paintings found in the mural (Anak Tomb no. 3, during Goguryeo Dynasty in 357 AD) support cattle existence in Korean peninsula. However, there are several reports about cattle originating in a controversial way [4]. Whatever their origin, JJBC has been categorized as an endangered species due to a substantial shrinkage in population size until 1980s. It is reported that registered JJBC comprise approximately 619 individuals (Korea Seed Stock Database), while other sources indicated that the population size might be 400 to 500 [5,6]. Due to the small size of the JJBC population, it is essential to evaluate and monitor the level of inbreeding, which is an important parameter to assess the genetic diversity of the breed.

JJBC are adapted to the subtropical environment of the Jeju Island. Also, beef of JJBC is rich in oleic acid, linoleic acid, and unsaturated fatty acids, which make it a premium quality for Korean consumers. However, in the past decades, this indigenous breed was paid little attention by beef cattle producers due to their slow growth and thus were was not competitive compared to ‘Hanwoo’, a breed in mainland of Korea that has been extensively bred for superior meat quality.

Genomic studies using high throughput whole genome sequencing data have become popular in recent years. Single nucleotide polymorphisms (SNP) are one of the common genetic variants for any organism, and genotyping with a high density SNP microarray chip provides genome information in an efficient and cost effective manner. Many useful genetic parameters such as linkage disequilibrium (LD), effective population size (N_e_), inbreeding coefficient, levels of heterozygosity, etc. can be estimated with high density SNP chips [7–9], so as to provide powerful tools to study evolutionary and conservation biology.

Development of sustainable breed improvement strategies depends on the precise characterization of animal genetic diversity [10]. Although several studies have investigated the diversity pattern of Korean cattle along with JJBC [2,5,11], it is still controversial whether Jeju Black are a separate breed. Thus, an accurate definition of breed origin for JJBC is necessary for future conservation and improvement programs. Genetic diversity study with microsatellite markers often causes estimated values of greater genetic differentiation than SNP markers [12,13]. Moreover, SNP markers give more accurate estimates in population admixture analysis than pedigree information [14]. In this study, genetic characteristics and population structure of JJBC breed were investigated with high density SNP chips, and were compared with Hanwoo, seven *Bos indicus* and *Bos taurus* breeds, by analyzing allelic richness (A_R_), level of inbreeding (F_IS_), effective population size (N_e_), and genetic distances between the breeds.

## MATERIALS AND METHODS

### Animals and genotypes

Animal ethics approval statement was not applicable as the Hanwoo DNA was extracted either from commercial bull semen straws or from tail hair samples obtained from different farmers with the permission of the owners. A total of 373 animals from nine cattle breeds were chosen to study, including two indicine breeds (Brahman, 15; Nelore, 26), seven taurine breeds (Angus, 27; Holstein, 76; Hereford, 25; Hanwoo, 66; Jeju Black, 78; Brown Wagyu, 10; and Black Wagyu, 50). Hanwoo DNAs were extracted either from AI bull semen straws or tail hair samples obtained from different farmers with the permission of the owners, and JJBC DNAs were prepared either from AI bull semen straws or tail hair in Jeju Island, Korea. Holstein DNAs were collected from semen straws provided by Nonghyup Dairy cattle improvement center. All other cattle DNAs were provided by Texas A & M University, USA, except the two Japanese Wagyu breeds. No ethics statement was required for the collection of DNA samples from the Brahman, Nelore, Angus, and Hereford cattle, because DNA samples were provided by the two authors in USA under their rules and regulations. The data of two Japanese Wagyu breeds were provided by the authors in Japan, which was previously used and published. Genomic DNA purification and genotyping was accomplished by DNA Link Inc. (Seoul, Korea), a commercial genome analysis service provider in Korea.

### Single nucleotide polymorphism genotyping and assembly of data sets

The samples of all breeds in this study except the two Wagyu breeds were genotyped using a customized Affymetrix 150K SNP Axiom array by DNA Link Inc. (Seoul, Korea), which included the 50K SNPs from the Bovine 50K v.3 Bead Chip (Illumina, San Diego, CA, USA). The samples of the two Wagyu breeds were genotyped with the Illumina 50K SNP chip. All the genotyped data were then merged and the common SNP markers on autosomal chromosomes were selected, resulting in 45,526 SNP markers in the final dataset across the breeds.

### Quality control and filtering of single nucleotide polymorphism markers

SNP quality control and filtering were performed across the nine cattle breeds to remove SNP markers with less than 95% call rate and animals with less than 95% call rate with PLINK software program [15]. This process resulted in 41,186 SNPs in 372 animals across the breeds. SNP quality filtering was also performed using PLINK version 1.9, and SNP markers with high LD were pruned using the following parameter option; -indep pair wise command--*indep-pairwise 50 5 2* (SNP window size, 50; SNP markers shifted per step, 5; *r*^2^ threshold, 2), because pruning of SNP markers in high LD can counter the effect of ascertainments bias and generate meaningful comparison between breeds [16]. After pruning, a total of 18,524 SNP markers were finally selected for analysis.

### Estimation of genetic diversity within breed and population differentiation

Genetic diversity within cattle breeds can be estimated by expected heterozygosity (H_E_), observed heterozygosity (H_O_), and inbreeding coefficient (F_IS_), for which the parameters were calculated using R software package divRsity v1.9.9 [17]. Analysis of molecular variance (AMOVA) to determine the partition of genetic diversity was performed with ARLEQUIN v3.5 [18]. Population differentiation was calculated by pairwise F_ST_ estimates according to the approach of Weir and Cockerham’s [19] with R package, divRsity v1.9.9.

### Population structure analysis

Population structure of the studied cattle breeds were also carried out using the software ADMIXTURE v1.3. [20], which enables an unsupervised clustering of large numbers of samples and allows the incorporation of each individual cattle breed into a mixture of clusters. In ADMIXURE, a model-based estimation of individual ancestry was applied for a range of prior values of K defined by the user. To elicit the true number of genetic populations, i.e. K clusters among nine cattle breeds; a cross validation (CV) approach was used to determine the most likely number of populations (K) in the SNP data. The best possible number of ancestral populations (K) was inferred through 3 to 11 pre-assumed populations. For each tested value of K in ADMIXURE, the proportion of each individual’s genotype was estimated for clustering, showing a preferable value of K with a low CV error compared with other K values.

### Principal component analysis

Principal component analysis (PCA) determines breed relationships that are based directly on allele frequencies by using a multivariate method, in which the information from a large number of alleles and loci is condensed into a few synthetic variables known as principal components (PC) [21]. PCA was carried out to infer relationships between the nine cattle populations by using PLINK v1.9.

### Genetic distance

Phylogenic analysis on the basis of SNP data has become an important tool for studying evolutionary history of any organism. In this study, phylogenic tree was constructed by calculating Nei’s genetic distances (DA) [22] with Poptree 2 [23] program. Neighbor-Joining (NJ) method was applied for measuring Nei’s genetic distance (D_A_), which can be defined as

$$D_{A}=1-\frac{1}{r}\sum_{j}^{r}\sum_{i}^{m_{j}}\sqrt{x_{ij}y_{ij}}$$

where *x_ij_* and *y_ij_* are the frequencies of the *i*-th allele at the *j*-th locus in populations X and Y, respectively, *m_j_* is the number of alleles at the *j*-th locus, and *r* is the number of loci used.

### Linkage disequilibrium

To measure the extent of LD, Lewontin’s D′ [24] and Hill’s *r*^2^ [25] are widely used. However, *r*^2^ is preferred for association studies due to its robustness, simplicity and not sensitivity to changing gene frequency and effective population size [26]. The *r*^2^ estimator represents a squared correlation coefficient (r) between two variables (alleles) at two separate SNP marker loci [27]. PLINK software [15] was used for estimation of *r*^2^ parameter:

$$r^{2}(P_{a},P_{b},P_{ab})=\left(\frac{{\left(P_{ab}-P_{a}P_{b}\right)}^{2}}{P_{a}(1-P_{a})P_{b}(1-P_{b})}\right)$$

where, *P_ab_* represents the frequency of haplotypes consisting of allele *a* at the first locus and allele *b* at the second locus [28]. Ther2 values were calculated for each chromosome in each breed, as well as across breeds, so that correlations between all possible SNPs were tested and there was no *r*^2^ threshold, in order to encapsulate all possible linkage interaction between SNPs per chromosome. To display the decay of LD, distances of pair-wise SNPs were binned into twenty types of intervals (0 to 1 kb, 10 kb intervals starting from 1kb up to 100 kb and 100 kb interval starting from 100 kb up to 1 Mb). For each chromosome, *r*^2^ values were then sorted by inter-SNP distance, and were averaged across the afore-mentioned intervals to observe possible *r*^2^ patterns with increasing inter-SNP distances.

### Effective population size (N**_e_**)

N_e_ was estimated using SNeP v1.1 by Barbato et al [29], which was based on LD data with the following formula suggested by Corbin et al [30],

$$N_{T(t)}={\left(4f(c_{t})\right)}^{-1}\;\left(E{\left[r_{adj}^{2}|c_{t}\right]}^{-1}-\alpha \right),$$

where *N_T(t)_* represents the past effective population size with *t* generations ago, *C_t_* represents the recombination rate, *t* generations ago in the past for specific physical distance between markers calculated by the SNeP tool, *r^2^_adj_* represents LD value adjusted for sample size, and α = (1, 2, 2.2) is the correction factor (constant) for the occurrence of mutation. The recombination rate was calculated using the following equation suggested by Sved [31],

$$f(\text{c})=c[(1-c/2)/{\left(1-c\right)}^{2}]$$

The data sets for each sub-population, as well as the merged dataset, were grouped into 20 distance bins of 10 to 100 kb each. N_e_ estimates were subsequently obtained from the *r*^2^ values for the average distance of each distance bin.

## RESULTS

### Within breed genetic diversity

Table 1 presents three measures of within breed diversity across the studied population. The minor allele frequency (MAF) ranged from 0.11 (Nelore) to 0.21 (Hanwoo, Angus, and Holstein), while the MAF was in middle (0.16) in JJBC. Nelore cattle had the lowest level of expected heterozygosity (H_E_ = 0.15), while Hanwoo, Angus, and Holstein had the highest level of genetic diversity (H_E_ = 0.28). JJBC had a middle range value of H_E_ (0.21) in the studied breeds.

In a structured population, the fixation index, F represents the degree of reduction in heterozygosity relative to Hardy-Weinberg expectation. F_IS_ measures the heterozygosity of individuals (I) relative to the subpopulation (S) represented by non-random mating (inbreeding), so that negative F_IS_ value means less inbreeding. The F_IS_ ranged from −0.018 in Black Wagyu to −0.118 in Brown Wagyu cattle. JJBC had the F_IS_ value of −0.076, while −0.025 in Hanwoo (Table 1).

### Analysis of molecular variance and population differentiation

**Figure 1:**
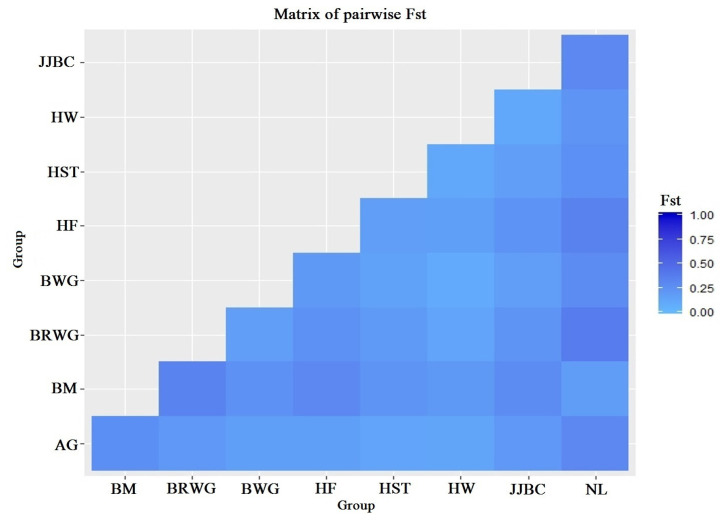
Graphical representation of pairwise F_ST_ distance matrix. AG, Angus; BM, Brahman; BRWG, Brown Wagyu; BWG, Black Wagyu; HF, Hereford; HST, Holstein; HW, Hanwoo; JJBC, Jeju Black cattle; NL, Nelore.

The average Wright’s F-statistics that were estimated with 20,000 bootstraps over loci were of values, F_ST_ = 0.173, F_IS_ = −0.030 and F_IT_ = 0.148 (Table 2). F_IT_ measures the genetic differentiation within individuals of the total population. F_ST_ is the measure of the genetic differentiation between breeds, for which the value of 0.173 indicates population differentiation with statistical significance (p<0.01). AMOVA indicated that almost 17% of the variation was estimated for variation among the populations, while 85% of the variation was accounted due to within individual variation (Table 3). Genetic differentiations between the nine cattle breeds that were based on pairwise F_ST_ are displayed in Figure 1. The F_ST_ ranged from 0.085 (Hanwoo and Brown Wagyu) to 0.376 (Nelore and Brown Wagyu). The genetic distance (D_A_) between Hereford and Nelore (0.107) was relatively greater than between JJBC and Hanwoo (0.028). JJBC had great distances with Nelore (0.084) and Brahman (0.078), while small distances with Hanwoo (0.028) and Black Wagyu (0.042) cattle breeds.

**Figure 2:**
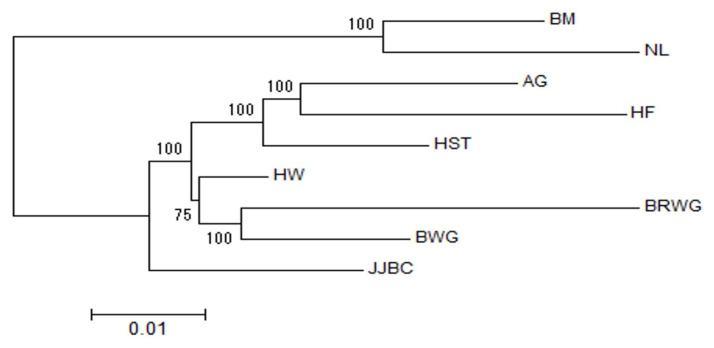
Unrooted consensus tree showing the genetic relationships among the nine breeds using the neighbor-joining method and the unbiased Nei’s D_A_ genetic distance. The values at the nodes are the percentages of bootstrap values from 1,000 replications of resampling. AG, Angus; BM, Brahman; BRWG, Brown Wagyu; BWG, Black Wagyu; HF, Hereford; HST, Holstein; HW, Hanwoo; JJBC, Jeju Black cattle; NL, Nelore.

The Nei’s genetic distances (D_A_) matrix of the nine cattle breeds were also used to construct phylogenetic trees with NJ method [22]. Our results showed clear separation of taurine and indicine cattle into two groups (branches). After diverging into two branches of *Bos Taurus* and *Bos Indicus*, the NJ trees showed two sub-branches within the taurine branch, i.e. one for JJBC and the other for Hanwoo, two Japanese breeds and three European taurine breeds (Figure 2).

### Principal component analysis

**Figure 3:**
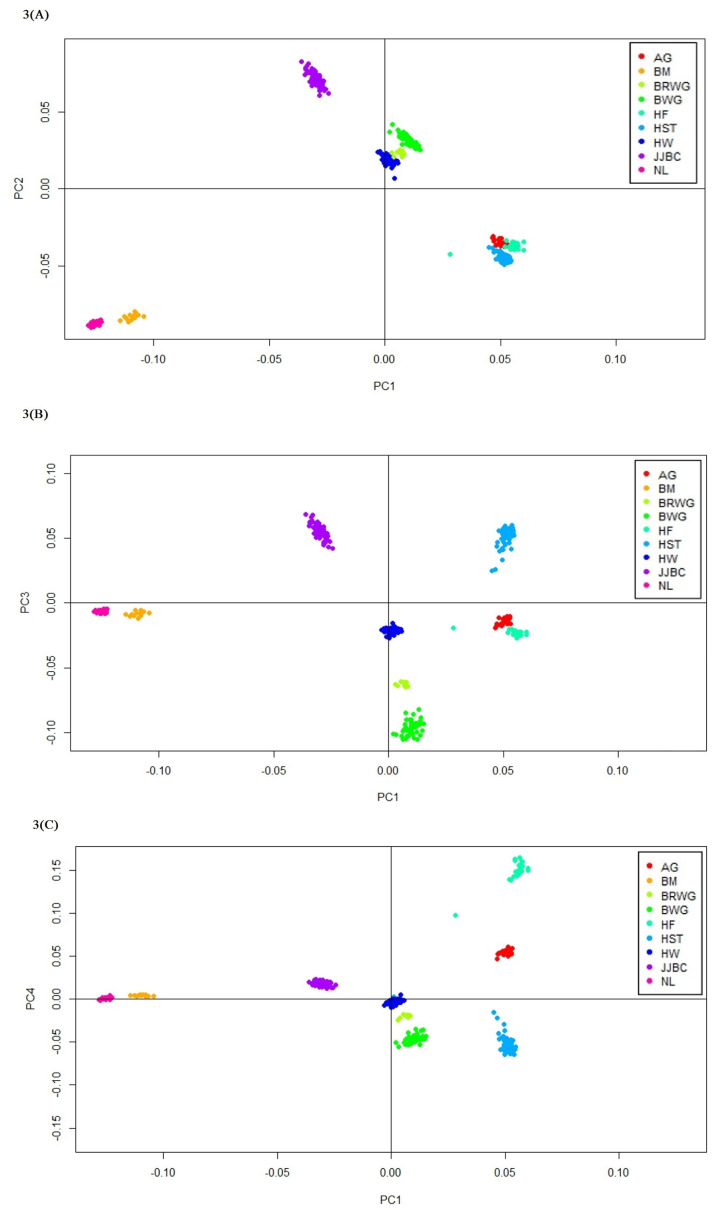
Principal component analysis (PCA) analyses. First and second principal component (A), first and third principal component (B), and first and fourth principal component (C) analysis resulted from the 18,524 single nucleotide polymorphisms (SNPs) in the nine cattle breeds. AG, Angus; BM, Brahman; BRWG, Brown Wagyu; BWG, Black Wagyu; HF, Hereford; HST, Holstein; HW, Hanwoo; JJBC, Jeju Black cattle; NL, Nelore.

The first and second PC accounted for 27.9% and 24.9% of the total variation respectively, while the third and fourth PC accounted for 19.0% and 16.7% of the total variation, respectively. Thus, the first five PC accounted almost 100% variation across the breed populations. JJBC were uniquely located, even if Hanwoo breed is more closely positioned to JJBC than the other cattle breeds (Figure 3). Nelore and Brahman formed a distinct cluster due to large variation between taurine and indicine cattle. Hanwoo cattle formed a closer cluster with Brown Wagyu and Black Wagyu than Holstein or other European taurine breeds (Figure 3).

### Population structure analysis between eight cattle breeds

**Figure 4:**
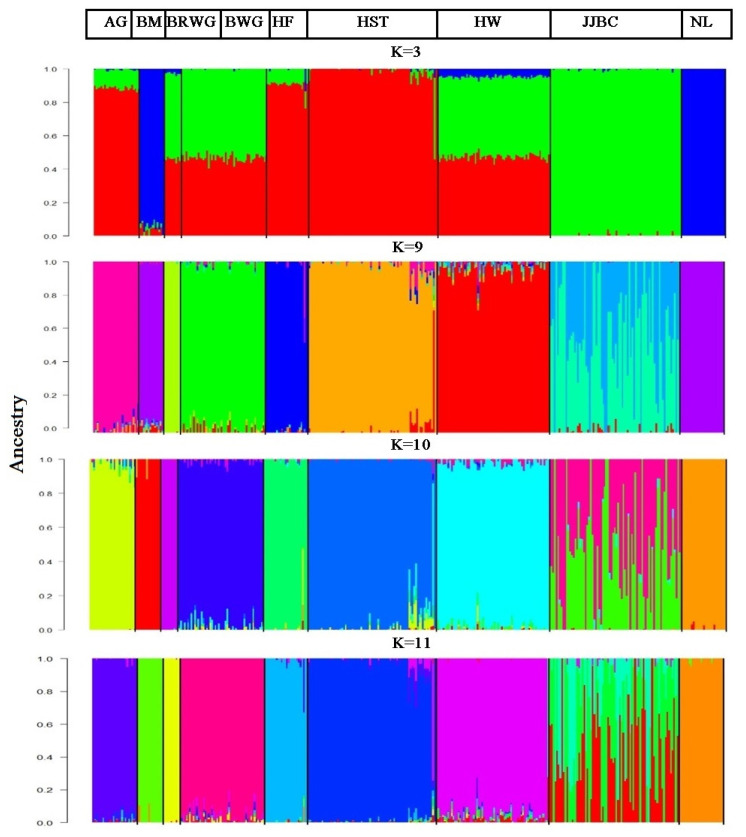
Clustering assignments of individuals into the nine cattle populations. ADMIXTURE analysis were performed with inferred K values ranging from 2 to 15, while the clustering results were shown when k = 3, 9, 10 and K = 11. AG, Angus; BM, Brahman; BRWG, Brown Wagyu; BWG, Black Wagyu; HF, Hereford; HST, Holstein; HW, Hanwoo; JJBC, Jeju Black Cattle; NL, Nelore. Each individual was represented by a single vertical line, which was divided into K colored segments. K is thenumber of the clusters assumed to have proportional length to each of the K inferred clusters. Black color separates the populations. Breeds are labeled by abbreviation at the top of figure.

The results of proportion of individuals into each of the nine breeds that were inferred by the ADMIXTURE are presented in Figure 4. The lowest CV error values were expected when K = 9. However, the lowest CV error estimator was found when K = 11 (data not shown). Thus, K = 11 was taken as the most probable number of inferred populations.

### Effective population size (N**_e_**) over the past generations

**Figure 5:**
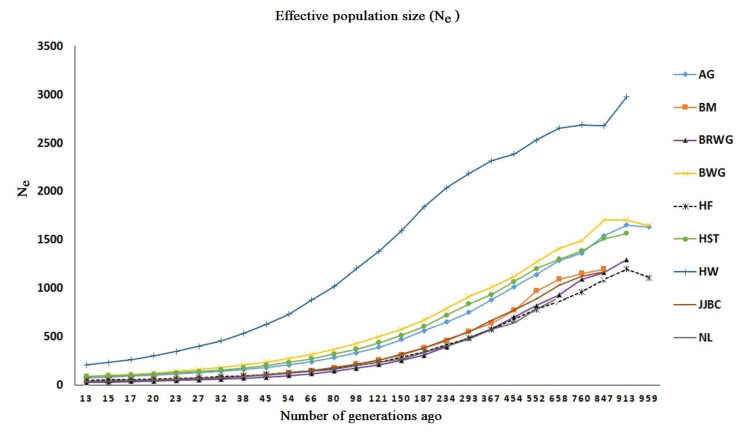
Effective population size (N_e_) in the nine cattle breeds. AG, Angus; BM, Brahman; BRWG, Brown Wagyu; BWG, Black Wagyu; HF, Hereford; HST, Holstein; HW, Hanwoo; JJBC, Jeju Black cattle; NL, Nelore.

N_e_ is needed to determine the accuracy of genomic selection [28], and the N_e_ estimates in the nine cattle breeds are shown in Figure 5 and Table 4 at *t* generation ago. In general, all the nine breeds showed a marked decrease over time as expected. The N_e_ estimate of Hanwoo cattle in the most recent 13 generation ago was 209, which was the greatest value across the nine breeds, while JJBC has an N_e_ size of 38 at the 13 generation ago, which was small compared with other cattle breeds, e.g. 78, 37, 39, 51, 29, 93, and 91 for Angus, Brahman, Nelore, Hereford, Brown Wagyu, Black Wagyu, and Holstein, respectively.

### Linkage disequilibrium

LD measurements with *r*^2^ in the nine cattle breeds are presented in the Supplementary Table S1. In general, cattle breeds showed different pattern of LD at different inter-SNP distances (Supplementary Table S2). The average *r*^2^ value for JJBC was 0.71 at 0 to 1 kb distance bin, which was very high compared with other cattle breeds, i.e. Hanwoo, Brown Wagyu, Black Wagyu, Brahman, Nelore, Angus, Hereford, and Korean Holstein had the *r*^2^ values of 0.63, 0.65, 0.54, 0.55, 0.58, 0.71, 0.63, and 0.67, respectively. The highest LD within close proximity of 0 to 1 kb interval distance decreased rapidly with increasing distance between pairs of SNP markers in all populations.

## DISCUSSION

Genetic characterization of breeds or animals based on genomic data has become an attractive method due to easy access of high throughput data derived from microarray SNP chip technology. In genetic diversity analysis, SNP markers have many advantages over microsatellite markers due to higher level of resolution, despite a set of microsatellites being suggested by the FAO to assess genetic diversity of farm animals and endangered species [10,32]. In the most recent years, genomic characterization using SNP markers have been studied in a variety of cattle breeds such as Irish Carry cattle [33], Tyrol Grey [34], Spanish beef cattle breeds [35], Canchim [36], Chinese Yiling yellow cattle [37] and many other indigenous and exotic cattle breeds raised in different countries worldwide [38–44].

Among Korean cattle breeds, Hanwoo was paid much more attention due to its incorporation into the national breeding program since 1970s [2]. In this study, we emphasized on the genetic characterization of JJBC, because these cattle are raised in Jeju Island in Korea and thus have unique characteristics that are different from the breeds on the mainland of Korea.

JJBC showed lower level of genetic variability (H_E_ = 0.21) than Hanwoo and Holstein in Korea (H_E_ = 0.218). Sharma et al [11,45] demonstrated a different level of heterozygosity in JJBC (H_E_ = 0.39 and 0.25), while Struken et al [2] reported H_E_ = 0.29. Heterozygosity level in our study is close to Sharma et al [11,45]. However, different results might be due to the use of various genotyping platforms, markers, and quality control criteria [2]. The lower level of heterozygosity in JJBC than Hanwoo and Holstein might be due to small population sizes, or few breeding males with more chance for increased inbreeding. However, Makina et al [40] stated that allele frequencies might be a poor estimate of inbreeding.

Inbreeding level (F_IS_) in JJBC was estimated to be −0.076, which was lower than Hanwoo (−0.025) and Holstein (−0.026) in Korea. Genetic variability was lowest in Nelore (H_E_ = 0.15) and low in Brahman (H_E_ = 0.17). Indicine breeds might have less genetic variability than taurine breeds as reported by Lin et al [46]. Analysis of molecular variance that enables partitioning of genetic variation into overall fixation indices (F_ST_), within population inbreeding (F_IS_) and total inbreeding (F_IT_), showed that 85% of total genetic variation was due to within populations across nine cattle breeds. This value was lower than the within populations genetic variation observed in South African cattle populations (92%) [40], but higher than those for Iranian cattle (82.9%) [42] and Ethiopian cattle populations (84.0%) [39]. Total inbreeding estimate (F_IT_) and estimate of population differentiation (F_ST_) was0.148 and 0.173 respectively, with statistical significance (p<0.001) (Table 2). JJBC and Hanwoo were found to be least differentiated (F_ST_ = 0.106) compared toother breed pairs. Sharma et al [45] calculated F_ST_ values for Korean cattle breeds, ranging 0.02 to 0.06, andreported that JJBC was differentiated from another Korean cattle breed, Brindle Hanwoo (Chikso). Struken et al [2] estimated F_ST_ value of 0.024 for JJBC-Hanwoo, 0.038 for JJBC-Chikso and 0.023 for Hanwoo-Chikso. Bothstudies confirmed that JJBC was least differentiated with Hanwoo, which was in good agreement with our study.

Japanese breed Black Wagyu breeds showed less differentiation with Hanwoo (0.085) than JJBC (0.164), indicating a closer relation with Hanwoo. Based on F_ST_ value and phylogenetic tree, we are in agreement with the concept that Hanwoo and Japanese Wagyu cattle breeds were genetically much closer to each other than other indicine and European taurine breeds (Table 1; Figure 2). PCA analysis also showed that Hanwoo and Wagyu breeds aremuch closer than JJBC (Figure 3A).

Unsupervised hierarchical clustering of our data implemented with ADMIXTURE analysis revealed that 95% of Hanwoo individuals were assigned to cluster ten, whereas 39%, 38%, 23%, and 1% of JJBC individuals were assigned to four different clusters, i.e. five, one, six, and ten, respectively. This means that JJBC shared its genome only with Hanwoo cattle. Japanese Brown Wagyu (100%) stands alone in cluster three, while Black Wagyu (96%) individuals were assigned in cluster eleven and the rest of the 4%were assigned to cluster three, nine and ten (Table 4). This level of admixture between JJBC and Hanwoo and between Brown Wagyu and Black Wagyu might be due to co-ancestry regarding the origin of these two breeds.

Phylogenic tree analysis also showed a distinct branch for JJBC, while Hanwoo and Wagyu breeds shared the same branch more recently than JJBC (Figure 2), suggesting that Hanwoo and Wagyu breeds descended from a more close common ancestors than JJBC, and that JJBC was evolutionally diverged for a longer time than the breeds in inland Korea and Japan. This result supports that JJBC has been adapted in Jeju island, to have genetically unique characteristics as one cattle breed. PCA results also showed that Hanwoo individuals formed close clusters with those of the two Wagyu breeds than the JJBC individuals (Figure 3A), supporting the results of phylogenic tree analysis (Figure 2).

JJBC have an effective population size of 38 in the nearest 13 generation ago, whereas Sharma et al [11] reported the N_e_ estimate of 67. Sudrajad et al [5] reported that N_e_ in JJBC was estimated to be 60 until 11 generation ago, and Struken et al [2] reported the N_e_ estimate of 11 at nearest generation. These N_e_ estimates were much smaller than the N_e_ of Hanwoo, i.e. 209. Differences of N_e_ estimates in the various reports might be caused by many factors such as sample size, SNP quality control measures and models used to study LD and N_e_ [5].

The small N_e_ value of JJBC seems to be sufficient for maintaining genetic diversity for short term species management as suggested by Frankham et al [47], but not enough at long term level. This result indicates that careful implementation of breed conservation is needed to keep genetic diversity of JJBC while increasing the population, as well as in a breeding program for genetic improvement of economically important traits in beef cattle.

## CONCLUSION

This study supports genetic uniqueness of JJBC breed that has been evolved differently from Hanwoo and Wagyu breeds, as well as from indicine and western taurine breeds, although a small amount of genetic components in terms of allele sharing exists between JJBC and Hanwoo. However, further in-depth study with whole genome sequencing and scanning using high density markers with larger samples would help us to accurately measure genetic parameters for establishment of JJBC as a unique breed.

## Figures and Tables

**Table 1:** Sample sizes and measurements of genetic diversity for nine cattle breeds

Breed	N	MAF (SD)	H_O_ (SD)	H_E_ (SD)	F_IS_
Angus	27	0.21(0.16)	0.30(0.20)	0.28(0.18)	−0.056
Brahman	15	0.12(0.15)	0.18(0.22)	0.17(0.19)	−0.073
Brown Wagyu	10	0.16(0.17)	0.25(0.24)	0.22(0.20)	−0.118
Black Wagyu	50	0.19(0.16)	0.26(0.20)	0.26(0.19)	−0.018
Hereford	25	0.19(0.16)	0.27(0.21)	0.26(0.19)	−0.045
Holstein	75	0.21(0.16)	0.28(0.19)	0.28(0.18)	−0.026
Hanwoo	66	0.21(0.16)	0.29(0.19)	0.28(0.18)	−0.025
Jeju Black	78	0.16(0.16)	0.23(0.21)	0.21(0.19)	−0.076
Nelore	26	0.11(0.15)	0.16(0.21)	0.15(0.19)	−0.059

N, sample size; MAF, means of minor allele frequency; SD, standard deviation; H_O_, observed heterozygosity; H_E_, expected heterozygosity under Hardy-Weinberg equilibrium; F_IS_, inbreeding coefficient.

**Table 2:** Average F-Statistics overall loci according to Weir and Cockerham [20]

Fixation indices	Ninebreeds	p-values
F_ST_	0.173^**^	0.00000
F_IS_	−0.030^NS^	1.00000
F_IT_	0.148^**^	0.00000

F_ST_, genetic differentiation among breeds; F_IS_, within population inbreeding; F_IT_, total inbreeding; NS, not significant.

** p<0.001 significant levels were obtained after 1,000 permutations. Fixation indices were obtained with over 20,000 bootstraps.

**Table 3:** Analysis of molecular variance among the nine cattle breeds

Data set	Variance components (%)		

	Among populations	Among individuals within populations	Within individuals
All nine cattle breeds	17.31^**^	−2.48^NS^	85.17^**^

** p<0.001significant levels were obtained after 1,000 permutations; NS, not significant.

**Table 4:** Effective population size (N_e_) across the nine cattle breeds

Generation Ago	AG	BM	BRWG	BWG	HF	HST	HW	JJBC	NL
13	78	37	29	93	51	91	209	38	39
15	85	42	33	102	55	97	235	42	43
17	94	47	37	113	59	106	264	47	49
20	102	53	41	126	64	115	306	53	53
23	114	59	47	142	70	128	347	61	60
27	128	67	54	161	77	142	401	68	68
32	142	78	61	183	87	158	459	79	78
38	161	93	71	208	97	179	536	90	88
45	182	108	83	238	111	205	628	107	105
54	210	125	99	275	128	236	732	127	121
66	242	150	120	318	147	273	880	151	141
80	284	178	144	369	170	321	1,020	181	166
98	336	217	174	427	202	371	1,205	216	201
121	393	263	212	503	239	438	1,383	260	238
150	471	313	255	578	290	515	1,595	326	271
187	562	385	308	671	344	604	1,839	386	336
234	652	460	394	789	416	723	2,035	467	402
293	753	554	488	917	484	840	2,188	551	474
367	881	639	586	1,014	574	937	2,318	668	575
454	1,016	771	707	1,121	683	1,068	2,386	775	646
552	1,141	971	823	1,272	781	1,203	2,532	891	787
658	1,286	1,092	933	1,408	863	1,300	2,653	1,031	903
760	1,364	1,151	1,093	1,492	962	1,386	2,686	1,125	-
847	1,544	1,200	1,162	1,703	1,088	1,514	2,678	1,168	-
913	1,653	-	1,297	1,708	1,200	1,567	2,978	-	-

AG, Angus; BM, Brahman; BRWG, Brown Wagyu; BWG, Black Wagyu; HF, Hereford; HST, Holstein; HW, Hanwoo; JJBC, Jeju Black cattle; NL, Nelore.
