# Supplementary material for: Genetic characteristics of Korean Jeju Black cattle with high density single nucleotide polymorphisms
Source: Anim Biosci. 2020 Aug 21;34(5):789–800. doi: 10.5713/ajas.19.0888 (PMC8100474; doi:10.5713/ajas.19.0888)
Supplement: Supplementary file 1 [file ajas-19-0888-suppl.pdf]

Table S1. Linkage disequilibrium (LD), expressed as r^2 and genetic distance between SNPs in different generations ago in the nine cattle breeds

Gen Ago	AG		BM		BRWG		BWG		HF		HST		HW		JJBC		NL	
	r^2	SD	r^2	SD	r^2	SD	r^2	SD	r^2	SD	r^2	SD	r^2	SD	r^2	SD	r^2	SD
**13**	0.08	0.10	0.16	0.18	0.19	0.19	0.07	0.09	0.12	0.14	0.07	0.09	0.03	0.04	0.15	0.18	0.15	0.17
**15**	0.08	0.11	0.16	0.18	0.19	0.20	0.07	0.10	0.12	0.15	0.07	0.10	0.03	0.04	0.16	0.19	0.15	0.18
**17**	0.09	0.11	0.16	0.18	0.20	0.20	0.07	0.10	0.13	0.16	0.08	0.10	0.03	0.05	0.16	0.19	0.15	0.18
**20**	0.09	0.12	0.16	0.18	0.20	0.20	0.08	0.10	0.14	0.16	0.08	0.11	0.03	0.05	0.16	0.19	0.16	0.18
**23**	0.09	0.12	0.17	0.19	0.20	0.21	0.08	0.11	0.15	0.17	0.09	0.11	0.03	0.05	0.16	0.19	0.17	0.19
**27**	0.10	0.12	0.17	0.19	0.21	0.21	0.08	0.11	0.15	0.18	0.09	0.12	0.03	0.05	0.17	0.20	0.17	0.19
**32**	0.10	0.13	0.17	0.19	0.21	0.21	0.08	0.11	0.16	0.18	0.09	0.12	0.03	0.05	0.17	0.20	0.17	0.19
**38**	0.11	0.13	0.17	0.19	0.21	0.21	0.09	0.11	0.17	0.19	0.10	0.12	0.03	0.05	0.18	0.20	0.18	0.20
**45**	0.11	0.14	0.18	0.20	0.22	0.21	0.09	0.12	0.17	0.19	0.10	0.13	0.04	0.05	0.18	0.21	0.18	0.20
**54**	0.12	0.14	0.18	0.20	0.22	0.22	0.09	0.12	0.18	0.20	0.10	0.13	0.04	0.05	0.18	0.21	0.19	0.20
**66**	0.12	0.15	0.18	0.20	0.22	0.22	0.09	0.13	0.18	0.20	0.11	0.14	0.04	0.05	0.18	0.21	0.19	0.21
**80**	0.12	0.15	0.18	0.21	0.22	0.22	0.10	0.13	0.19	0.21	0.11	0.14	0.04	0.05	0.18	0.21	0.19	0.21
**98**	0.13	0.15	0.19	0.20	0.22	0.22	0.10	0.14	0.20	0.22	0.12	0.15	0.04	0.06	0.19	0.21	0.20	0.21
**121**	0.13	0.16	0.19	0.21	0.22	0.22	0.11	0.14	0.20	0.22	0.12	0.15	0.04	0.06	0.19	0.22	0.20	0.22
**150**	0.14	0.16	0.19	0.21	0.23	0.22	0.12	0.15	0.21	0.22	0.13	0.16	0.05	0.07	0.19	0.22	0.22	0.23
**187**	0.14	0.17	0.20	0.21	0.23	0.22	0.12	0.16	0.21	0.23	0.13	0.16	0.05	0.07	0.20	0.22	0.22	0.23
**234**	0.15	0.18	0.20	0.22	0.23	0.22	0.13	0.17	0.22	0.23	0.14	0.17	0.05	0.08	0.20	0.23	0.23	0.23
**293**	0.16	0.19	0.21	0.22	0.23	0.23	0.14	0.17	0.23	0.24	0.15	0.18	0.06	0.10	0.21	0.23	0.24	0.24
**367**	0.17	0.20	0.22	0.23	0.24	0.23	0.15	0.19	0.24	0.25	0.16	0.20	0.07	0.11	0.22	0.24	0.24	0.24
**454**	0.18	0.21	0.23	0.23	0.24	0.23	0.17	0.21	0.25	0.25	0.18	0.21	0.09	0.13	0.23	0.25	0.26	0.25
**552**	0.20	0.21	0.22	0.23	0.25	0.24	0.18	0.21	0.26	0.26	0.19	0.21	0.10	0.15	0.24	0.26	0.26	0.25
**658**	0.20	0.22	0.23	0.23	0.26	0.24	0.19	0.23	0.28	0.27	0.20	0.23	0.11	0.16	0.24	0.26	0.27	0.26
**760**	0.22	0.23	0.25	0.25	0.26	0.24	0.20	0.23	0.28	0.27	0.22	0.24	0.12	0.18	0.25	0.27	-	-
**847**	0.22	0.23	0.26	0.25	0.27	0.24	0.20	0.23	0.28	0.27	0.22	0.24	0.14	0.18	0.27	0.27	-	-
**913**	0.22	0.23	-	-	0.26	0.24	0.21	0.25	0.28	0.26	0.23	0.26	0.13	0.19	-	-	-	-
**959**	0.23	0.24	-	-	-	-	0.23	0.25	0.30	0.28	-	-	-	-	-	-	-	-

Gen Ago, Generation ago; AG (Angus), BM (Brahman), BRWG (Brown Wagyu), BWG (Black Wagyu), HF (Hereford), HST (Holstein), HW (Hanwoo), JJBC (Jeju Black Cattle), NL (Nelore).

Table S2. Mean linkage disequilibrium (LD), r2 estimates for different distances between syntenic SNPs estimated with PLINK v1.9

Distance Interval (kb)	r^2 (Mean ± SD)									
	**AG**	**BH**	**BRWG**	**BWG**	**HF**	**HST**	**HW**	**JJBC**	**NL**	**Total**
0-1	0.71±0.36	0.55±0.42	0.65±0.4	0.54±0.42	0.63±0.42	0.67±0.36	0.63±0.36	0.71±0.34	0.58±0.39	0.63±0.39
1-10	0.43±0.38	0.40±0.39	0.48±0.4	0.41±0.38	0.47±0.40	0.39±0.38	0.30±0.34	0.42±0.40	0.39±0.37	0.41±0.38
10-20	0.33±0.35	0.34±0.34	0.42±0.37	0.32±0.36	0.38±0.37	0.31±0.34	0.23±0.29	0.33±0.36	0.36±0.39	0.34±0.35
20-30	0.31±0.32	0.32±0.35	0.43±0.37	0.28±0.33	0.38±0.36	0.28±0.32	0.20±0.27	0.30±0.34	0.31±0.35	0.31±0.33
30-40	0.27±0.30	0.30±0.33	0.37±0.35	0.24±0.30	0.33±0.34	0.24±0.29	0.16±0.23	0.26±0.32	0.30±0.34	0.27±0.31
40-50	0.25±0.29	0.31±0.34	0.36±0.34	0.22±0.28	0.31±0.33	0.23±0.28	0.15±0.21	0.24±0.31	0.28±0.33	0.26±0.30
50-60	0.23±0.28	0.28±0.31	0.34±0.33	0.20±0.26	0.30±0.32	0.20±0.26	0.12±0.19	0.23±0.30	0.27±0.32	0.24±0.29
60-70	0.22±0.26	0.28±0.32	0.33±0.33	0.19±0.25	0.28±0.31	0.19±0.25	0.12±0.18	0.22±0.29	0.26±0.31	0.23±0.28
70-80	0.21±0.26	0.27±0.31	0.33±0.32	0.17±0.24	0.27±0.30	0.18±0.24	0.10±0.16	0.21±0.27	0.26±0.32	0.22±0.27
80-90	0.19±0.23	0.26±0.30	0.30±0.31	0.16±0.23	0.25±0.29	0.17±0.22	0.09±0.15	0.19±0.26	0.24±0.30	0.21±0.25
90-100	0.19±0.23	0.26±0.31	0.30±0.30	0.15±0.21	0.26±0.29	0.16±0.22	0.08±0.14	0.19±0.25	0.23±0.30	0.20±0.25
100-200	0.16±0.21	0.24±0.29	0.28±0.29	0.13±0.19	0.22±0.26	0.14±0.19	0.06±0.11	0.17±0.24	0.21±0.28	0.18±0.23
200-300	0.14±0.18	0.21±0.27	0.26±0.27	0.11±0.16	0.20±0.24	0.12±0.16	0.05±0.08	0.15±0.22	0.18±0.24	0.16±0.20
300-400	0.13±0.17	0.20±0.26	0.25±0.26	0.10±0.14	0.18±0.22	0.11±0.15	0.04±0.07	0.15±0.21	0.17±0.23	0.15±0.19
400-500	0.12±0.16	0.20±0.25	0.24±0.26	0.09±0.14	0.18±0.22	0.10±0.14	0.04±0.06	0.14±0.20	0.16±0.22	0.14±0.18
500-600	0.12±0.15	0.19±0.25	0.24±0.25	0.09±0.13	0.17±0.21	0.10±0.14	0.04±0.06	0.14±0.20	0.15±0.21	0.14±0.18
600-700	0.11±0.15	0.19±0.25	0.23±0.25	0.08±0.13	0.17±0.21	0.09±0.13	0.03±0.05	0.14±0.20	0.15±0.21	0.13±0.18
700-800	0.11±0.14	0.19±0.24	0.23±0.25	0.08±0.12	0.16±0.20	0.09±0.13	0.03±0.05	0.14±0.20	0.14±0.20	0.13±0.17
800-900	0.11±0.14	0.19±0.24	0.23±0.25	0.08±0.12	0.16±0.20	0.09±0.13	0.03±0.05	0.14±0.20	0.14±0.20	0.13±0.17
900-1000	0.11±0.14	0.19±0.24	0.23±0.25	0.08±0.12	0.15±0.19	0.09±0.12	0.03±0.05	0.13±0.19	0.14±0.20	0.13±0.17
**Total**	0.22 ±0.24	0.27 ±0.30	0.33 ±0.31	0.19±0.23	0.27 ±0.28	0.20 ±0.24	0.13 ±0.16	0.23 ±0.27	0.25 ±0.29	-

AG (Angus), BM (Brahman), BRWG (Brown Wagyu), BWG (Black Wagyu), HF (Hereford), HST (Holstein), HW (Hanwoo), JJBC (Jeju Black Cattle), NL (Nelore).
